# Single nucleotide polymorphisms associated with elevated alanine aminotransferase in patients receiving asunaprevir plus daclatasvir combination therapy for chronic hepatitis C

**DOI:** 10.1371/journal.pone.0219022

**Published:** 2019-07-10

**Authors:** Keizo Kato, Noritomo Shimada, Masanori Atsukawa, Hiroshi Abe, Norio Itokawa, Yoshihiro Matsumoto, Rie Agata, Akihito Tsubota

**Affiliations:** 1 Liver Disease Control Science, Graduate School of Organic Pathology and Therapeutics, The Jikei University School of Medicine, Minato-ku, Tokyo, Japan; 2 Division of Gastroenterology and Hepatology, Shinmatsudo Central General Hospital, Matsudo, Chiba, Japan; 3 Division of Gastroenterology and Hepatology, Ootakanomori Hospital, Kashiwa, Chiba, Japan; 4 Division of Gastroenterology and Hepatology, Department of Internal Medicine, Nippon Medical School, Bunkyo-ku, Tokyo, Japan; 5 Division of Gastroenterology, Department of Internal Medicine, Nippon Medical School Chiba Hokusoh Hospital, Inzai, Chiba, Japan; 6 Division of Gastroenterology and Hepatology, The Jikei University School of Medicine, Kashiwa Hospital, Chiba, Japan; 7 Core Research Facilities, Research Center for Medical Sciences, The Jikei University School of Medicine, Minato-ku, Tokyo, Japan; Nihon University School of Medicine, JAPAN

## Abstract

**Aims:**

Drug-induced liver damage characterized by serum alanine aminotransferase (ALT) elevation often occurs in direct-acting antiviral (DAA) combination therapy for chronic hepatitis C virus (HCV) infection. This study explored single nucleotide polymorphisms (SNPs) at drug metabolism- or transport-related genes that were associated with ALT elevation in asunaprevir plus daclatasvir therapy.

**Methods:**

Subjects were 185 Japanese patients with chronic HCV genotype 1b infection who received asunaprevir plus daclatasvir therapy. Tag SNPs at possible metabolizing enzyme and transporter genes, which were involved in the pharmacokinetics of asunaprevir and daclatasvir, were selected.

**Results:**

Among the tag SNPs analyzed, *CYP3A4* rs4646437 was significantly associated with ALT elevation (p = 0.013): maximum ALT values in patients with genotype CC were higher than those in patients with genotype non-CC (allele T). The proportion of grades 2–4 in genotype CC patients were significantly greater than those in genotype non-CC patients (p = 0.028). No patients with genotype non-CC showed grade ≥2 ALT elevation. In multivariate analysis, rs4646437 genotype CC and cirrhosis were significant, independent factors associated with grade ≥1 ALT elevation (odds ratio, 2.83 and 1.88; p = 0.040 and 0.045, respectively). In exploratory analyses, although serum concentrations of asunaprevir and daclatasvir were not correlated with maximum ALT values or rs4646437 genotypes, asunaprevir concentrations in patients with grade ≥1 ALT elevation were significantly higher than those in patients with grade <1 ALT elevation (P = 0.023).

**Conclusions:**

*CYP3A4* rs4646437 was found to be significantly and independently associated with ALT elevation in Japanese patients receiving ASV plus DCV therapy. Notably, none of the patients with rs4646437 genotype non-CC (allele T) had grade ≥2 ALT elevation. SNP genotyping prior to treatment might be useful for carefully monitoring patients to complete treatment safely.

## Introduction

Antiviral therapy for chronic hepatitis C virus (HCV) infection has advanced dramatically in recent years. Interferon-based treatment yields a low rate of HCV elimination with frequent and varied adverse events and poor tolerability, whereas combinations of two or three different direct-acting antiviral (DAA) classes produce an extremely high rate of viral elimination with fewer and milder adverse events, and good tolerability. Currently, interferon-free, dual or triple oral DAA combination therapy is the standard of care for HCV infection. Asunaprevir [ASV; nonstructural protein (NS) 3/4A protease inhibitor] plus daclatasvir (DCV; NS5A replication-complex inhibitor) therapy is the first, interferon-free, DAA treatment regimen available for chronic HCV genotype 1 infection in Japan. This treatment has been available since September 2014, and yields a sustained virological response (SVR) rate of approximately ~90% [1–6].

Dual therapy was, or is still used in several Latin American and Asian countries (including Japan, China, and South Korea), and triple therapy with ASV, DCV, and beclabuvir (nonnucleoside NS5B thumb-1 polymerase inhibitor) is available in some countries [7–9]. However, these treatment regimens often induce liver damage which is characterized by elevation of serum alanine aminotransferase (ALT), aspartate aminotransferase (AST), and bilirubin levels [2, 3, 9]. Both ASV and DCV are absorbed rapidly via passive permeability and as substrates for P-glycoprotein (P-gp), which is involved in an efflux mechanism. ASV and DCV are transported exclusively to the liver through active uptake by organic anion transporting polypeptide (OATP)-mediated transport and organic cation and undefined transporters, respectively. Both ASV and DCV are metabolized primarily by cytochrome P450 family 3 subfamily A member 4 (CYP3A4)-mediated hepatic oxidative reactions [10–13].

Due to the highly preferential hepatic disposition, ALT elevation is the most common laboratory abnormality; ALT elevation of grade ≥3 is observed in 5.1–12.6% of Japanese patients and frequently results in treatment discontinuation [2–6]. In general, NS3/4A protease inhibitors, including ASV, are more closely associated with drug-induced liver damage compared to other DAA classes [10, 13–15]. Furthermore, the pharmacokinetic profile is influenced by the activity of major metabolizing enzymes (such as CYP3A4) and transporters (such as OATP and P-gp) [10, 16, 17]. Given that DAA regimens, including next-generation NS3/4A protease inhibitors such as grazoprevir and glecaprevir, are currently available, it is important to elucidate the factors associated with drug-induced liver damage. Since a relationship between serum ASV concentrations and ALT elevation has been suggested in Japanese patients [6,18], the difference in expression or activity of metabolizing enzymes and transporters in the liver, or single nucleotide polymorphisms (SNPs) at the coding genes, could be involved in the pharmacokinetics and underlying mechanisms for drug-induced liver damage [5].

The aim of this retrospective study was to investigate factors associated with drug-induced ALT elevation in patients receiving ASV plus DCV for chronic HCV infection. Specifically, this study focused on the relationship between ALT elevation and SNPs at *CYP3A4*, *CYP3A5*, *OATP1B1*, *OATP2B1*, and *P-gp*, which are involved in the pharmacokinetics of ASV and DCV (i.e., absorption from the intestine, active uptake into hepatocytes, intrahepatic metabolism, and excretion into bile canaliculus) [10–13]. In addition, exploratory analyses were performed to evaluate the correlation between these SNPs, ALT elevation, and serum concentrations of ASV and DCV.

## Patients and methods

### Study population and design

One hundred and eighty-five patients with chronic HCV genotype 1b infection were enrolled in this study at Shinmatsudo Central General Hospital, Nippon Medical School Chiba Hokusoh Hospital, Ootakanomori Hospital, and Jikei Kashiwa Hospital between September 2014 and July 2016. Patients received an oral dose of 100 mg ASV (Bristol-Myers Squibb, New York, NY, USA) twice daily and 60 mg DCV (Bristol-Myers Squibb) once daily for 24 weeks. Leading inclusion criteria were as follows: 1) diagnosis of chronic HCV infection through laboratory and virology findings; 2) HCV genotype 1b confirmed by the conventional polymerase chain reaction (PCR)-based method; 3) age ≥20 years; 4) acquisition of written informed consent from each individual; and 5) availability of genetic DNA for genotyping SNPs. Leading exclusion criteria included 1) decompensated liver cirrhosis (Child-Pugh class B or C); 2) other forms of liver disease, such as alcoholic liver disease, autoimmune hepatitis, and primary biliary cirrhosis; 3) malignant tumors including hepatocellular carcinoma under treatment; 4) concurrent treatment with any contraindicated drugs; 5) positive for hepatitis B surface antigen or human immunodeficiency virus; 6) concurrent treatment with any other antiviral or immunomodulatory agent; 7) hypersensitivity to ASV or DCV; and 8) pregnancy or lactation. Physical, hematological, and biochemical examinations were performed at treatment initiation, every 2 weeks during treatment period, and each post-treatment visit (post-treatment weeks 4, 8, 12, and 24) during the 24-week follow-up period. Fibrosis-4 (FIB-4) index was also calculated, as previously reported [19]. Serum samples were stored at -80˚C until use. The design of this study was complied with the ethical guidelines of the Helsinki Declaration (2013) and was approved by the ethics committee of each institution [Institutional Review Board of The Jikei University School of Medicine-approved No. 29–003 (8619)].

### Virology and definition of treatment responses

Serum HCV RNA levels were measured using a real-time PCR method with the lower quantification limit of 1.2 log IU/mL (COBAS TaqMan HCV Test 2.0; Roche Diagnostics, Tokyo Japan). SVR was defined as undetectable HCV RNA at 24weeks post-treatment. Relapse was defined as undetectable HCV RNA at the end of treatment but detectable viremia during the follow-up period. Non-virological response was defined as persistent viremia throughout the treatment. Viral breakthrough was defined as undetectable HCV RNA after treatment but reappearance of HCV RNA during the treatment, or as an increase in the HCV RNA level of ≥1.0 log IU/mL from the lowest value during treatment period. Variants at L31 and Y93 in the NS5A region of HCV genome were detected using the direct sequencing method. The amino acid sequences of L31 and Y93 were defined as wild type.

### Grading ALT elevation

ALT elevation after treatment initiation could reflect drug-induced liver damage and might cause treatment cessation, which often occurs in Japanese patients treated with ASV plus DCV [2–6]. In this study, the Division of Acquired Immunodeficiency Syndrome criteria [20] were used to evaluate the severity of ALT elevation. The maximum ALT value after a minimum recorded on-treatment value was <1.25 times the upper limit of normal (ULN) for grade 0, 1.25–2.5 × ULN for grade 1 (mild), 2.6–5.0 × ULN for grade 2 (moderate), 5.1–10.0 × ULN for grade 3 (severe), and >10.0 × ULN for grade 4 (potentially life-threatening).

### Selection and genotyping of SNPs

SNPs at *CYP3A4*, *CYP3A5*, *OATP1B1*, *OATP2B1*, and *P-gp*, with minor allele frequencies >0.1 among the Japanese population, were selected based on the HapMap Japanese in Tokyo database. Among these, tag SNPs were further determined using PLINK version 1.9 and Haploview version 4.2 software (Broad Institute, Cambridge, MA, USA). Pairwise linkage disequilibrium statistics, estimated frequencies of haplotype, and haplotype blocks were also calculated using these software packages. Hardy–Weinberg equilibrium was assessed using chi-square test with one degree of freedom. In addition, uridine diphosphate glucuronosyltransferase family 1 member A1 (*UGT1A1*) rs 4148323 was determined, because this SNP is reportedly associated with ASV/DCV-induced ALT elevation [5].

Genomic DNA was extracted from whole blood using the MagNA Pure LC and the DNA Isolation Kit (Roche Diagnostics). Tag SNPs were genotyped by real-time detection PCR using the TaqMan SNP Genotyping Assays or rhAmp SNP Genotyping System (IDT, Coralville, IA, USA) and the LightCycler 96 System (Roche Diagnostics).

### Serum concentrations of ASV and DCV

ASV and DCV concentrations were measured in serum samples from 17 patients with grade ≥1 ALT elevation and 18 patients with grade <1ALT elevation. These patients were selected randomly using a computer-generated random number list. Serum samples from patients with ALT elevation were analyzed using those obtained at the time of maximum elevation (median, 12 weeks after treatment initiation). Serum samples from patients without ALT elevation were assayed using those obtained 12 weeks after treatment initiation. Serum concentrations of ASV and DCV were measured using validated liquid chromatography with tandem mass spectrometry methods in Kobe laboratory, CMIC Pharma Science Co., Ltd. (Kobe, Japan) [21, 22].

### Statistical analysis

Data are expressed as medians and ranges for quantitative variables and as frequencies and percentages for qualitative variables. Mann–Whitney *U* test for quantitative variables and Fisher’s exact test for qualitative variables were used to compare two groups. Multiple comparisons of continuous variables among multiple groups were performed using Kruskal–Wallis test, followed by Steel–Dwass post-hoc test. Cochran–Armitage test for trends was performed to assess the presence of an association) between a variable with two categories and a variable with multiple categories. Spearman’s rank correlation coefficient was used to evaluate the correlation between two continuous variables. The distribution of SNP genotypes was tested for deviation from Hardy–Weinberg equilibrium and exclusion of selection bias using chi-squared goodness-of-fit test. Univariate and multiple logistic regression analyses were performed to identify significant variables associated with ALT elevation and SVR: each quantitative variable was categorized into two groups by the median value. All statistical analyses were performed using Excel Statistics 2015 for Windows (SSRI, Tokyo). All p values for statistical tests were two-tailed and values of <0.05 were considered statistically significant.

## Results

### Patient characteristics

Baseline characteristics of the 185 patients are summarized in S1 Table. The subject group comprised 103 females and 82 males, with a median age of 72 years (range, 37–87 years). Seventy-nine (42.7%) patients underwent prior interferon-based treatment. However, none had previously received interferon-free DAA treatment. Seventy-eight (42.2%) patients had compensated liver cirrhosis. Twenty-four (13.0%) patients had previously undergone transarterial chemoembolization and/or radiofrequency ablation for the curative treatment of hepatocellular carcinoma.

### Treatment outcomes

Among the 185 patients, 147 (79.5%) achieved SVR, 14 (7.6%) underwent relapse, 20 (10.8%) experienced viral breakthrough, and one (0.5%) showed non-virological response. The remaining three patients were missing during the follow-up period and were regarded as those with non-SVR based on the intention-to-treat principle.

Twenty-seven (14.6%) patients discontinued treatment prematurely (median, 10 weeks; range, 2–19 weeks). Of these 27 patients, 13 discontinued treatment due to viral breakthrough, seven due to severe ALT elevation, two due to rash, two due to fever, one due to acute myocardial infarction, one due to depression, and one due to aortic valve stenosis. Rash disappeared within five days, and fever resolved three days after treatment cessation. Among the 14 patients who discontinued treatment without viral breakthrough, 11 (78.6%; seven ALT elevation, one rash, one fever, one acute myocardial infarction, and one depression) achieved SVR. One patient (7.1%; aortic valve stenosis) suffered relapse, one (7.1%; fever) showed non-virologic response, and one (7.1%; rash) was missing.

### Association between ALT elevation and SVR

Based on the degree of ALT elevation, 113 patients (61.1%) were categorized as grade 0, 49 (26.5%) as grade 1, 11 (5.9%) as grade 2, seven (3.8%) as grade 3, and five (2.7%) as grade 4. Of the total 185 patients, seven (3.8%) discontinued treatment prematurely due to ALT elevation; these included 3/11 (27.3%) grade 2 patients, 1/7 (14.3%) grade 3, and 3/5 (60.0%) grade 4 (median, 17 weeks after treatment; range, 3–19 weeks; p = 1.48 × 10^−6^ by Cochran- Armitage test). Elevated ALT values were normalized 2–4 weeks after treatment cessation. As described above, all seven patients who discontinued treatment prematurely achieved SVR. The remaining 16 patients with grade ≥2 ALT elevation (eight grade 2, six grade 3, and two grade 4) completed the 24-week treatment course with a reduced dose of ASV and without modification of concurrent medication; of these 16 patients, 14 (87.5%) achieved SVR, one (6.3%) had relapse, and one (6.3%) experienced viral breakthrough. All patients with ALT elevation had ALT normalization or regression to the baseline level immediately after treatment completion or cessation, suggesting that ALT elevation occurred due to ASV plus DCV therapy.

**Fig 1:**
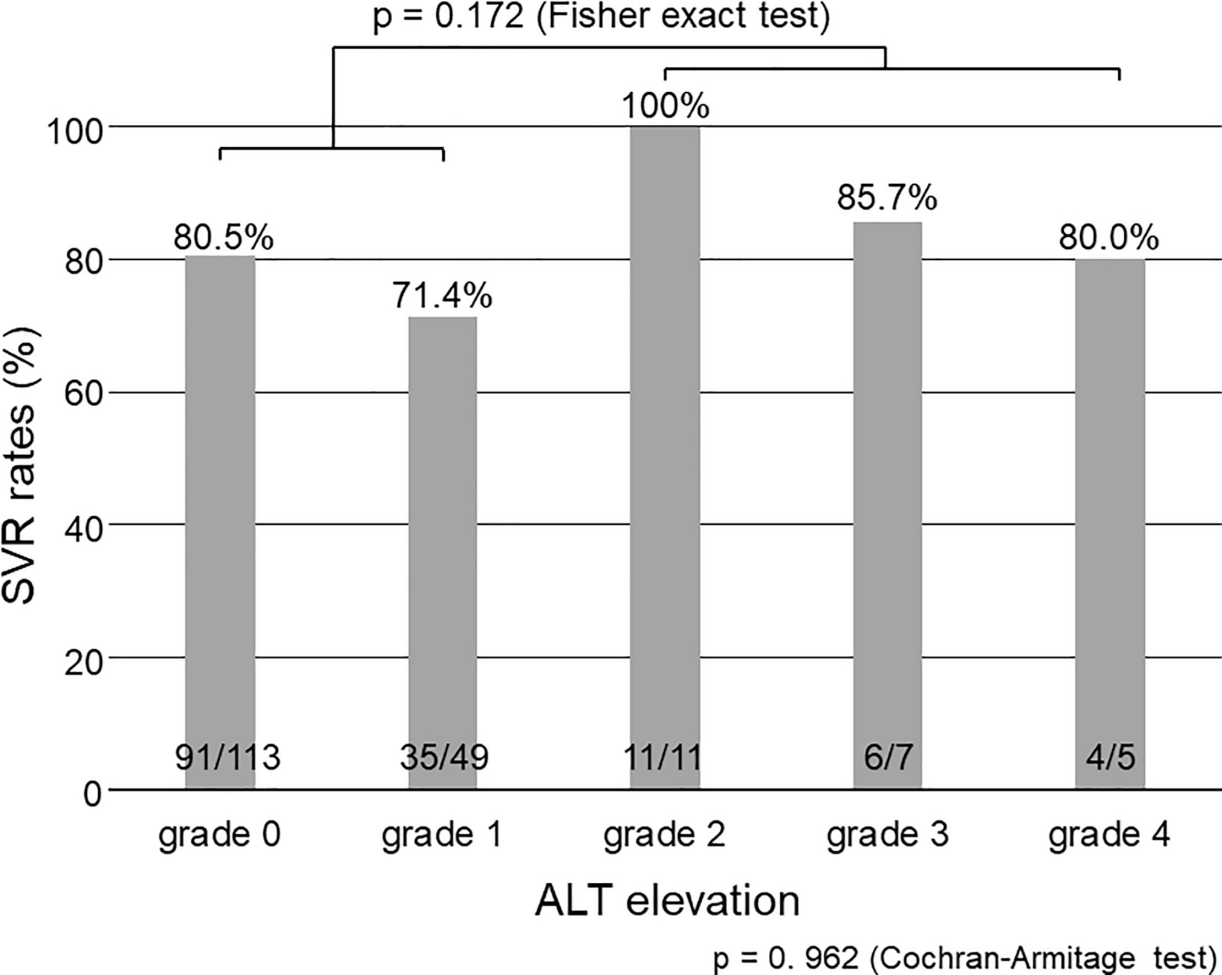
SVR rates according to the grade classification for ALT elevation. There is no significant association between grades of ALT elevation and SVR rates (p = 0.962 by Cochran–Armitage test for trends). The SVR rates in patients with grades 2–4 ALT elevation are numerically higher than those in patients with grades 0–1, although there is no significant difference (p = 0.172 by Fisher’s exact test). SVR, sustained virological response; ALT, alanine aminotransferase.

According to the degree of ALT elevation, the SVR rates were 80.5% (91/113) for grade 0, 71.4% (35/49) for grade 1, 100% (11/11) for grade 2, 85.7% (6/7) for grade 3, and 80.0% (4/5) for grade 4 (p = 0. 962 by Cochran-Armitage test; Fig 1). When patients were divided into two groups (grades 0–1 versus 2–4), the SVR rates were 77.8% (126/162) and 91.3% (21/23), respectively. The SVR rates in patients with grade 2–4 were numerically higher than those in patients with grades 0–1, although there was no statistical significance (p = 0.172).

### Association between ALT elevation and SNPs

We selected the following tag SNPs at each gene, as described in the Methods section: rs3735451, rs2246709, and rs4646437 at *CYP3A4*; rs776746 at *CYP3A5*; rs4149087,　rs4149064, rs4149048, rs7969341, and rs6487213 at *OATP1B1*; rs4944992, rs2712819, and rs11236365 at *OATP2B1*; and rs1202168, rs1922241, rs7779562, rs1858923, and rs868755 at *P-gp* (S2 Table). All tag SNPs were in Hardy–Weinberg equilibrium.

**Fig 2:**
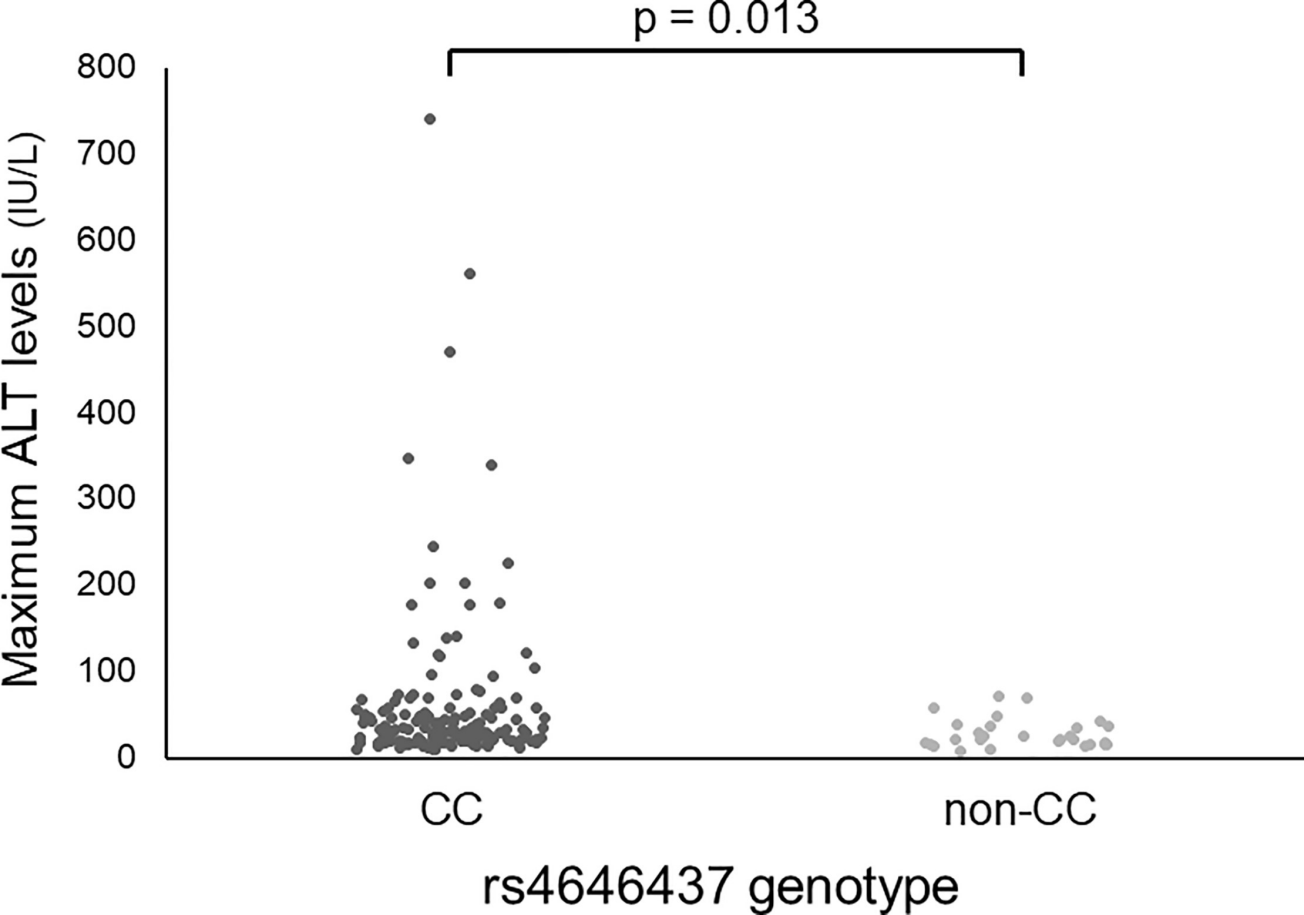
Maximum ALT values according to rs4646437 genotypes. Maximum ALT values in patients with genotype CC (median, 31 IU/mL; range, 9–740 IU/mL) are significantly higher than those in patients with genotype non-CC (median, 21.5 IU/mL; range, 8–71 IU/mL; p = 0.013 by Mann-Whitney *U* test). ALT, alanine aminotransferase.

**Fig 3:**
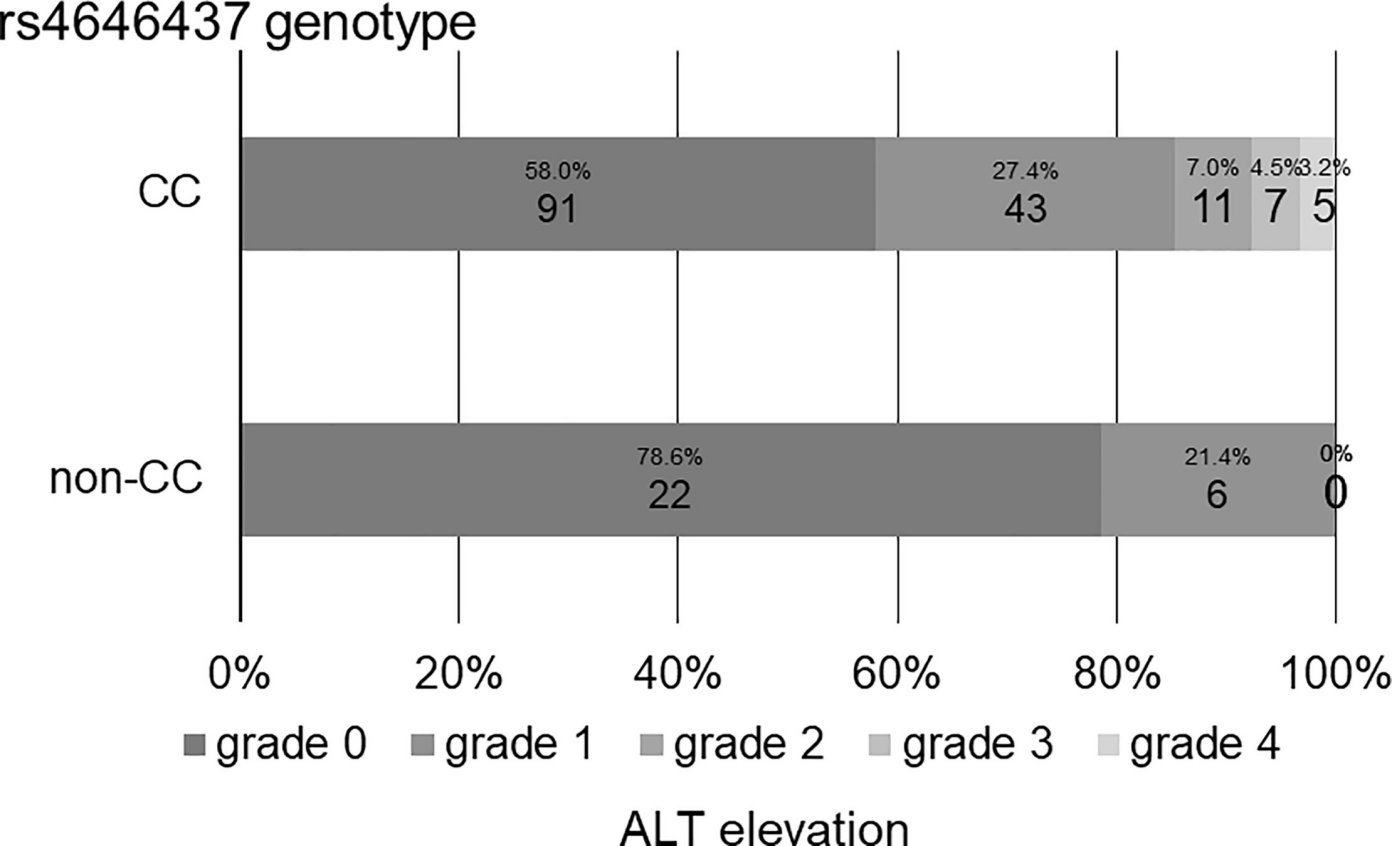
Grades of ALT elevation according to rs4646437 genotypes. Among 157 patients with rs4646437 genotype CC, 91 patients (58.0%) are grade 0, 43 (27.4%) are grade 1, 11 (7.0%) are grade 2, 7 (4.5%) are grade 3, and 5 (3.2%) are grade 4. Among 28 patients with rs4646437 genotype non-CC, 22 patients (78.6%) are grade 0, 6 (21.4%) are grade 1, and none (0%) are grades 2–4. The proportion of grades 2–4 in patients with genotype CC are significantly greater than those in patients with genotype non-CC (p = 0.028).

The association between ALT elevation and tag SNPs is shown in S3 Table. Among the tag SNPs analyzed, rs4646437 was significantly associated with ALT elevation (p = 0.013); the maximum ALT values in patients with rs4646437 genotype CC (median, 31 IU/mL; range, 9–740 IU/mL) were significantly higher than those in patients with genotype non-CC (median, 22 IU/mL; range, 8–71 IU/mL) (Fig 2). Among 157 patients with rs4646437 genotype CC, 91 (58.0%) were grade 0, 43 (27.4%) were grade 1, 11 (7.0%) were grade 2, seven (4.5%) were grade 3, and five (3.2%) were grade 4. Among 28 patients with rs4646437 genotype non-CC, 22 (78.6%) were grade 0, six (21.4%) were grade 1, and none (0%) were grades 2–4. The proportion of grades 2–4 in patients with genotype CC were significantly greater than those in patients with genotype non-CC (p = 0.028) (Fig 3). Intriguingly, all seven patients who discontinued treatment prematurely due to ALT elevation had rs4646437 genotype CC.

**Fig 4:**
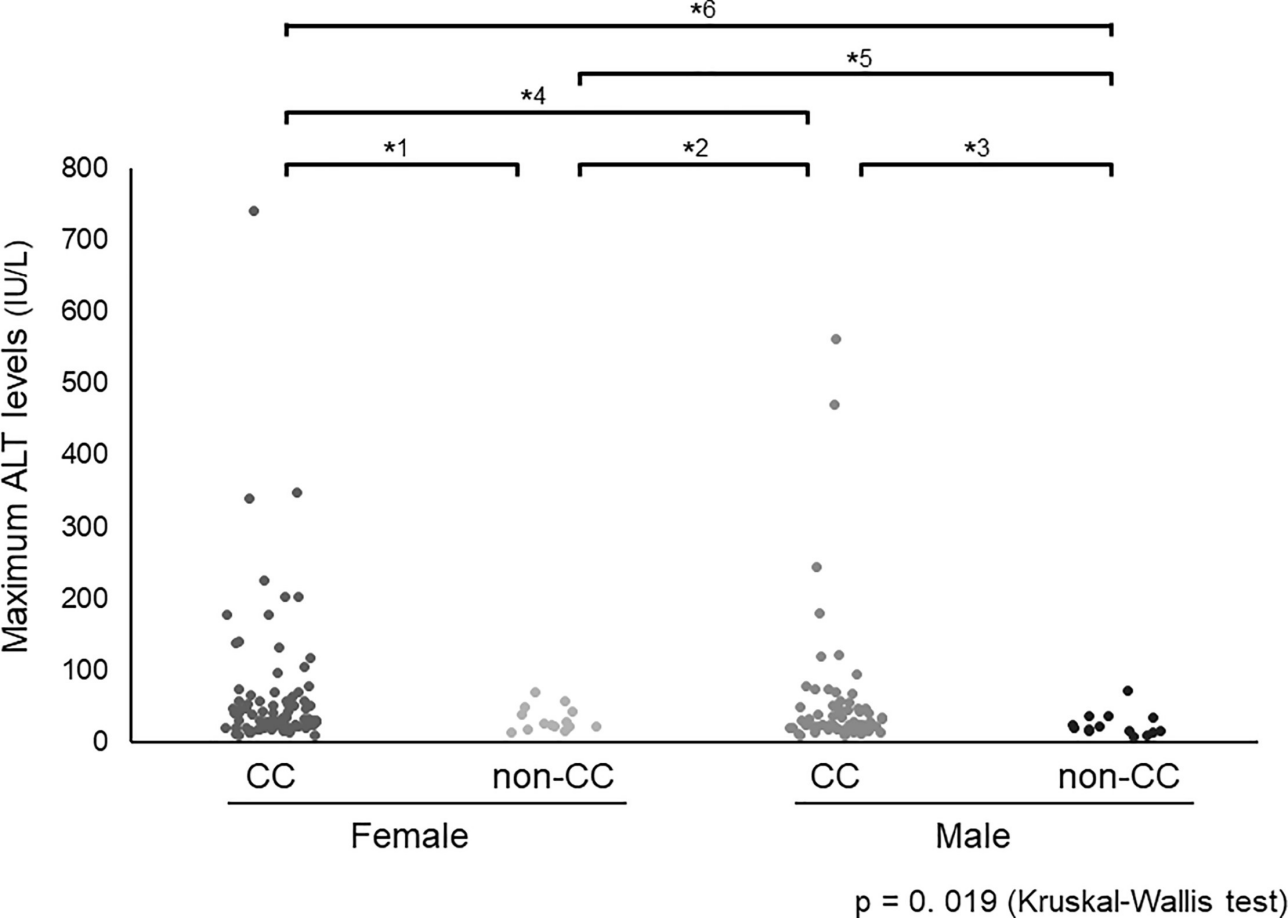
Maximum ALT levels according to gender and rs4646437 genotypes. The median of maximum ALT values is 33 IU/L (range, 9–740 IU/L) in females with rs4646437 genotype CC, 25.5 IU/L (range, 14–70 IU/L) in females with genotype non-CC, 25 IU/L (range, 9–561 IU/L) in males with genotype CC, and 19 IU/L (range, 8–71 IU/L) in males with genotype non-CC (p = 0.019 by Kruskal-Wallis test). Maximum ALT levels in males with genotype non-CC are significantly or marginally lower than those in females with genotype CC (p = 0.018) and males with genotype CC (p = 0.084; both by Steel-Dwass test). In contrast, females with genotype CC have a high propensity for ALT elevation, compared to other subgroups. *^1^p = 0.503, *^2^p = 0.976, *^3^p = 0.084, *^4^p = 0.460, *^5^p = 0.279, *^6^p = 0.018.

We also investigated the association between ALT elevation, gender, and rs4646437 genotype. The maximum ALT values in females (median, 32 IU/L; range, 9–740 IU/L) were marginally higher than those in males (median, 25 IU/L; range, 8–561 IU/L) (p = 0.055). The median of maximum ALT values was 33 IU/L (range, 9–740 IU/L) in females with rs4646437 genotype CC, 25.5 IU/L (range, 14–70 IU/L) in females with genotype non-CC, 25 IU/L (range, 9–561 IU/L) in males with genotype CC, and 19 IU/L (range, 8–71 IU/L) in males with genotype non-CC (Fig 4; p = 0.019 by Kruskal-Wallis test). The maximum ALT values in males with genotype non-CC were significantly or marginally lower than those in females (p = 0.018) and males (p = 0.084; both by Steel-Dwass test) with genotype CC. In contrast, females with genotype CC had a high propensity for ALT elevation, compared to other subgroups.

### Pretreatment factors associated with ALT elevation

Pretreatment factors associated with grade ≥1 ALT elevation were analyzed because no patient with rs4646437 genotype non-CC showed grade ≥2 ALT elevation (Fig 3). In univariate analysis, ALT elevation (grade ≥1) was significantly or marginally associated with the following factors: female gender (p = 0.074), liver cirrhosis (p = 0.043), rs4646437 genotype CC (p = 0.045), AST ≥46 IU/L (p = 0.070), and alpha-fetoprotein ≥5.9 ng/mL (p = 0.088). In the final step of multivariate analysis, rs4646437 genotype CC and cirrhosis were significant, independent factors associated with grade ≥1 ALT elevation [odds ratios, 2.83 and 1.88; 95% confidence intervals, 1.05–7.07 and 1.01–3.50; p = 0.040 and 0.045, respectively] (S4 Table). When the analysis was limited to patients with genotype CC (excluding genotype non-CC), there were no factors associated with ALT elevation.

### Factors associated with SVR

In univariate analysis, SVR was significantly associated with the following factors: absence of liver cirrhosis, pretreatment serum HCV RNA levels <6.2 log IU/mL, and HCV-NS5A Y93 wild type. In the final step of multivariate analysis, the same three factors were found to be significant, independent factors associated with SVR (S5 Table). ALT elevation and rs4646437 genotype were not significantly associated with SVR.

### Association between serum concentrations of ASV/DCV and ALT elevation

As described above, we measured ASV and DCV concentrations in serum samples from 17 patients with grade ≥1 ALT elevation and 18 patients with grade <1 ALT elevation. Serum concentrations of ASV and DCV were not correlated with elevated ALT values (p = 0.404 and 0.594; *r*_s_ = 0.146 and 0.093, respectively; S6 Table).

The median ASV concentration was 398.5 ng/mL (range, 23.8–5170 ng/mL) in patients with rs4646437 genotype CC and 352 ng/mL (range, 57.5–1200 ng/mL) in those with genotype non-CC (p = 0.888; S7 Table). The median DCV concentration was 823 ng/mL (range, 179–2830 ng/ml) in patients with rs4646437 genotype CC and 349 ng/mL (range, 263–1780 ng/ml) in those with genotype non-CC (p = 0.571).

Overall, these results from limited samples failed to show an association between serum concentrations of ASV/DCV and maximum ALT values or rs4646437 genotypes. However, serum ASV concentrations in patients with grade ≥1 ALT elevation were significantly higher than those in patients with grade <1 ALT elevation (P = 0023; S7 Table). Among other variables, only body mass index showed a marginal significant correlation with serum ASV concentrations (p = 0.052; *r*_s_ = 0.341; S6 and S7 Tables).

## Discussion

Serum aminotransferase elevation is the most frequent laboratory abnormality in chronic hepatitis C patients receiving ASV plus DCV therapy [2]. In a real-world cohort study [3], the incidence of grade ≥1 ALT elevation was 37.6% (245/651), which is similar to that obtained in the current study (38.9%; 72/185). Several previous studies have reported that the rates of grade ≥3 ALT elevation were 6.0% (39/651) [3], 7.2% (16/222) [2], 10.5% (26/247) [5], and 12.2% (34/278) [6], which are not significantly different from 6.5% (12/185) in the current study. The rates of treatment discontinuation due to ALT elevation, appearance time to ALT elevation and its peak, and rapid reversal of elevated ALT after treatment cessation were also similar among these different cohort studies. These similar profiles suggest that the results obtained from the current study are reasonable and might be true for other patient cohorts.

Drug-induced increases of liver-related enzymes, including ALT, are attributable mainly to ASV, rather than DCV [6, 10, 13, 15]. ASV increases the frequency of aminotransferase elevation in a dose-dependent manner [15]. Multivariate analysis identified serum ASV (but not DCV) concentrations as a significant, independent factor associated with grade ≥3 aminotransferase elevation [6]. In fact, a dose reduction of ASV enabled physicians to continue treatment without modifying the dose of DCV in patients with drug-induced liver damage [3, 4]. In general, other DAA classes, such as NS5A and NS5B inhibitors, cause liver damage less frequently compared to NS3/4A protease inhibitors. This may be attributable to their different motifs and physicochemical properties [10]. When assessing the relationship between SNPs and drug-induced liver damage in DAA regimens, including NS3/4A protease inhibitors, the focus should be on genes that affect the pharmacokinetics of NS3/4A protease inhibitors.

Among tag SNPs at *CYP3A4*, *CYP3A5*, *OATP1B1*, *OTAP2B1*, and *P-gp*, which are involved in the pharmacokinetics of ASV [10, 11], our study found that *CYP3A4* rs4646437 was the only significant, independent factor associated with ALT elevation. Specifically, the maximum ALT values in patients with rs4646437 genotype CC were significantly higher than those in patients with genotype non-CC (allele T). Notably, none of the patients carrying allele T displayed grade ≥2 ALT elevation and, therefore, did not discontinue treatment due to ALT elevation. This suggests that allele T may be protective against ASV-induced liver damage. However, because patients with genotype CC showed a wide range of ALT values, from normal to severely elevated, the SNP genotype alone could not determine who would develop moderate to severe ALT elevation. ASV has a complex pharmacokinetic profile indicating that there are unidentified individual factors, including SNPs not analyzed in the current study, that contribute more strongly or mutually to the pharmacokinetics and drug-induced liver damage [10].

According to the data presented in a recent study, one SNP was found to be associated with severe liver damage in both the derivation and confirmatory Japanese patient cohorts that received ASV plus DCV therapy. *UGT1A1* rs4148323 allele A was a significant risk factor for grade ≥3 ALT elevation (57% for AA, 18% for AG, and 4% for GG, P = 8.4 × 10^−6^) and drug discontinuation (22% for AA, 11% for AG, and 2.5% for GG, P = 8.7 × 10^−4^) [5]. However, our study failed to reconfirm a significant correlation for grade ≥3 ALT elevation (13% for AA, 3% for AG, and 7% for GG; P = 0.651) and drug discontinuation (0% for AA, 0% for AG, and 5% for GG; P = 0.145). *UGT1A1* is part of a complex locus that encodes various UDP-glucuronosyltransferase isoforms. *UGT1A1* rs4148323 allele A can reduce UDP-glucuronosyltransferase activity and impair metabolism of related substrates [5]. However, neither ASV nor DCV are likely to be substrates of UGT1A1 and definitively inhibit its enzymatic activity *in vivo*, although inhibition by both drugs was suggested *in vitro* [10, 12–14]. Meanwhile, both ASV and DCV are metabolized primarily by CYP3A4-mediated reactions and are inhibitors and inducers of this enzyme [10–14]. If universal SNPs associated with adverse events are determined, pretreatment SNP genotyping will be useful in completing treatment safely as scheduled.

As described above, ASV is eliminated primarily via CYP3A4-mediated oxidative metabolism [10,11]. rs4646437 is representative of *CYP3A4* SNPs, and numerous studies have reported its relationship with drug metabolism and adverse events in the treatment for various diseases: adverse events caused by efavirenz in human immunodeficiency virus [23] and sunitinib in metastatic renal cell carcinoma [24]; finasteride exposure in prostate cancer risk [25]; immune responses to antiretroviral therapy in human immunodeficiency virus [26]; voriconazole exposure in invasive fungal infections [27]; tacrolimus metabolism among Chinese transplant recipients [28,29]; and cyclosporine kinetics in Egyptian and White transplant recipients [30,31]. Reportedly, rs4646437 influences the protein expression and enzymatic activity of hepatic CYP3A4 in a sex-dependent manner; females have higher expression/activity compared to males. Furthermore, non-CC females have higher expression/activity than non-CC males, and slightly higher expression/activity than CC males [32]. Therefore, rs4646437 may be functionally relevant for CYP3A4 expression/activity. Intriguingly, rs4646437 exerts opposing effects in females and males. However, these findings contradict our present results, as in this study, females were found to have higher ALT values compared to males, while non-CC males, who might have the lowest expression/activity among sex-genotype subgroups, did not exhibit moderate-to-severe ALT abnormality.

There are several possible reasons for this inconsistency. Firstly, CYP3A4 expression/activity in subjects with each rs4646437 allele or genotype may differ between White and Japanese subjects. Secondly, CYP3A4 may produce ASV metabolites that cause liver damage. Lastly, other SNPs or enzymes may contribute to CYP3A4 expression/activity or metabolism. Measurements of CYP3A4 expression/activity in liver microsomes of Asian individuals, including Japanese, are needed to clarify the relationship between SNPs, CYP3A4 expression/activity levels, and adverse events.

Severe drug-induced liver damage has been reported frequently in Japanese patients, whereas it has been reported rarely in other ethnic groups [1–3,5–9]. Japanese subjects have higher ASV exposure compared to White subjects [10]. In contrast, Asians have slightly lower DCV exposure compared to Whites, but the pharmacokinetics is largely comparable between Japanese/Asians and Whites [13]. Therefore, it is conceivable that the difference in prevalence of drug-induced liver damage may arise from ethnic differences in ASV exposure and the SNPs at genes involved in the expression/activity of metabolizing enzymes and transporters of ASV.

According to the HapMap database for *CYP3A4* rs4646437, 79% of Japanese have genotype CC and 21% have non-CC (allele T), which may be protective against moderate-to-severe ALT elevation. There is a great diversity in the frequency of the protective allele T among different races: 17% in white Americans, 40% in Hispanics, and 85–100% in Black/Afro-Americans. In the Genome Aggregation Database and 1000 Genomes Project, the allele frequency is 9–10% in Europeans, 72–82% in Africans, and 16–17% in East Asians. However, one SNP (rs4646437) alone cannot elucidate the ethnic differences in ASV exposure and ALT elevation. Certain confounding variables (such as age, sex, and body weight), differences in OATP and/or CYP3A4 expression/activity, and hepatic blood flow may affect the pharmacokinetic difference among different populations. Race-specific distribution of haplotypes or known SNPs may not drive these differences, as neither the OATP1B1 haplotypes nor OATP2B1 SNPs correlate with pharmacokinetic parameters [10]. In contrast, intrinsic factors (such as age, sex, and ethnicity) and the severity of hepatic impairment do not impact DCV exposure and safety [13].

Increased ASV exposure is a predictor of drug-induced injury and correlates with increased AST/ALT/bilirubin, decreased albumin levels, and cirrhosis associated with lower ASV clearance [10]. In Japanese patients, high serum ASV concentrations are associated with severe ALT elevation [6,18], and the concentrations correlate positively with fibrosis markers and negatively with platelet count and albumin levels. Increased ASV exposure could arise from the severity of liver disease, which attenuates the hepatotropic disposition of ASV via reductions in CYP3A4-mediated metabolism, OATP transport, and hepatic blood flow [6,10,33]. Therefore, advanced liver fibrosis may be an important factor contributing to increased ASV concentrations and ALT elevation. In clinical practice, ASV is not recommended for patients with Child B/C, who have substantially increased ASV exposure [34], although mild liver impairment does not meaningfully affect the pharmacokinetics. The current study revealed that cirrhosis was significantly associated with ALT elevation, and that patients with grade ≥1 ALT elevation had higher serum ASV concentrations. However, the results failed to reconfirm the correlation of serum ASV concentrations with elevated ALT values or surrogate liver fibrosis markers, although the sample size was small. Collectively, increased ASV exposure is necessary but not enough for ALT elevation, and there are significant correlations between the severity of disease, ALT elevation, and ASV exposure.

There are several limitations to this study. First, this was a retrospective study including a small number of patients. Second, this study did not include a validation group to confirm the results. Third, we examined tag SNPs alone at a limited number of genes. Therefore, a large-scale genome wide association study, including both development and validation groups, is needed to determine the SNPs related to drug-induced liver damage.

In conclusion, *CYP3A4* (drug-metabolizing enzyme gene) rs4646437 was found to be significantly and independently associated with ALT elevation in Japanese patients receiving ASV plus DCV therapy. Notably, none of the patients with rs4646437 genotype non-CC (allele T) had grade ≥2 ALT elevation, and they did not prematurely discontinue treatment due to ALT elevation. SNP genotyping prior to treatment, particularly DAA regimens including NS3/4A protease inhibitors, might be useful for carefully monitoring patients to facilitate the completion of their treatment course safely.

## Supporting information

S1 Table Clinical characteristics of the patients who received asunaprevir and daclatasvir therapy for chronic hepatitis C. (DOCX)

S2 Table Frequency of tag SNP genotypes in patients who received asunaprevir/ daclatasvir combination therapy. (DOCX)

S3 Table Maximum ALT values in each tag SNP genotype of *CYP3A4*, *CYP3A5*, *OATP2B1*, *OATP1B1*, *P-gp*, and *UGT1A1*. (DOCX)

S4 Table Baseline factors associated with ALT elevation (grade ≥1). (DOCX)

S5 Table Factors associated with sustained virological response. (DOCX)

S6 Table Correlation of serum asunaprevir and daclatasvir concentrations with other quantitative variables on Spearman’s rank correlation coefficient. (DOCX)

S7 Table Comparison of serum asunaprevir and daclatasvir concentrations between two categories in each variable. (DOCX)
