# Supplementary material for: Single nucleotide polymorphisms associated with elevated alanine aminotransferase in patients receiving asunaprevir plus daclatasvir combination therapy for chronic hepatitis C
Source: PLoS One. 2019 Jul 10;14(7):e0219022. doi: 10.1371/journal.pone.0219022 (PMC6619746; doi:10.1371/journal.pone.0219022)
Supplement: S1 Table — (DOCX) [file pone.0219022.s001.docx]

**S1 Table.** Clinical characteristics of the patients who received asunaprevir and daclatasvir therapy for chronic hepatitis C.

| Age (years) | 72 (37–87) |
| --- | --- |
| Gender (female / male) | 103 / 82 |
| Body weight (kg) | 55.9 (34.0–96.0) |
| Body mass index (kg/m^2^) | 22.6 (16.0–31.0) |
| Cirrhosis (no / yes) | 107 / 78 |
| History of anti-HCV treatment (no / yes) | 106 / 79 |
| Prior anti-HCV treatment |  |
| IFN / peg-IFN | 4 / 3 |
| IFN / peg-IFN + RBV | 1/ 50 |
| Peg-IFN + RBV + TVR / SMV | 7 / 14 |
| History of therapy for HCC (no / yes) | 161 / 24 |
| White blood cells (/µL) | 4210 (1600–10400) |
| Hemoglobin (g/dL) | 13.2 (7.3–17.4) |
| Platelet count (×10^4^/μL) | 13.0 (3.9–34.5) |
| AST (IU/L) | 46 (11–227) |
| ALT (IU/L) | 40 (6–175) |
| γ-GTP (IU/L) | 39 (8–590) |
| Albumin (g/dL) | 4.0 (1.8–4.8) |
| Total bilirubin (mg/dL) | 0.69 (0.20–2.40) |
| Creatinine (mg/dL) | 0.74 (0.41–10.18) |
| α-fetoprotein (ng/mL) | 5.9 (1.3–157.8) |
| HCV RNA (log IU/mL) | 6.2 (3.0–7.4) |
| FIB-4 index | 3.92 (0.88–20.48) |
| NS5A L31 substitution (no / yes / unknown) | 146 / 6 / 33 |
| NS5A Y93 substitution (no / yes / unknown) | 138 / 14 / 33 |

Data expressed as medians and ranges in parentheses for continuous variables, and numbers for categorical variables. HCV, hepatitis C virus; IFN, interferon; peg-IFN, pegylated interferon; RBV, ribavirin; TVR, telaprevir; SMV, simeprevir; HCC, hepatocellular carcinoma; AST, aspartate aminotransferase; ALT, alanine aminotransferase; γ-GTP, γ-glutamyltransferase; NS5A, non-structural 5A.
