# Supplementary material for: Single nucleotide polymorphisms associated with elevated alanine aminotransferase in patients receiving asunaprevir plus daclatasvir combination therapy for chronic hepatitis C
Source: PLoS One. 2019 Jul 10;14(7):e0219022. doi: 10.1371/journal.pone.0219022 (PMC6619746; doi:10.1371/journal.pone.0219022)
Supplement: S2 Table — (DOCX) [file pone.0219022.s002.docx]

**S2 Table.** Frequency of tag SNP genotypes in patients who received asunaprevir/ daclatasvir combination therapy

| Tag SNP | Locus | Genotype | *n* | MAF | HWE |
| --- | --- | --- | --- | --- | --- |
| rs3735451 | *CYP3A4*, Intron 12 | TT/CT/CC | 105/69/11 | 0.246 | 0.940 |
| rs2246709 | *CYP3A4*, Intron 7 | AA/AG/GG | 79/81/25 | 0.354 | 0.561 |
| rs4646437 | *CYP3A4*, Intron 7 | CC/CT/TT | 157/27/1 | 0.078 | 0.890 |
| rs776746 | *CYP3A5*, Intron 3 | GG/AG/AA | 113/63/9 | 0.219 | 0.954 |
| rs4149087 | *OATP1B1*, 3’ UTR | GG/GT/TT | 94/73/18 | 0.295 | 0.491 |
| rs4149064 | *OATP1B1*, Intron 8 | AA/AG/GG | 80/86/19 | 0.335 | 0.557 |
| rs4149048 | *OATP1B1*, Intron 5 | AA/AG/GG | 76/79/30 | 0.376 | 0.223 |
| rs7969341 | *OATP1B1*, Intron 14 | AA/AG/GG | 77/80/28 | 0.368 | 0.342 |
| rs6487213 | *OATP1B1*, Intron 7 | TT/CT/CC | 81/83/21 | 0.341 | 0.882 |
| rs4944992 | *OATP2B1*, Intron 1 | TT/CT/CC | 53/103/29 | 0.435 | 0.071 |
| rs2712819 | *OATP2B1*, Intron 1 | TT/CT/CC | 81/88/16 | 0.324 | 0.246 |
| rs11236365 | *OATP2B1*, Intron 8 | AA/AG/GG | 59/96/30 | 0.422 | 0.384 |
| rs1202168 | *P-gp*, Intron 22 | TT/TC/CC | 71/83/31 | 0.392 | 0.425 |
| rs1922241 | *P-gp*, Intron 20 | CC/CT/TT | 86/85/14 | 0.305 | 0.259 |
| rs7779562 | *P-gp*, Intron 4 | CC/CG/GG | 72/88/25 | 0.373 | 0.817 |
| rs1858923 | *P-gp*, Intron 25 | TT/TC/CC | 63/89/33 | 0.419 | 0.872 |
| rs868755 | *P-gp*, Intron 20 | CC/CA/AA | 72/87/26 | 0.376 | 0.972 |
| rs4148323 | *UGT1A1*, Exon 1 | GG/GA/AA | 143/38/4 | 0.124 | 0.441 |

SNP, single nucleotide polymorphism; MAF, minor allele frequency; HWP, Hardy-Weinberg equilibrium; *CYP3A4*, cytochrome P450 family 3 subfamily A member 4; *CYP3A5*, cytochrome P450 family 3 subfamily A member 5; *OATP2B1*, organic anion transporting polypeptide 2B1; *OATP1B1*, organic anion transporting polypeptide 1B1; *P-gp*, P-glycoprotein; *UGT1A1*, uridine diphosphate glucuronosyltransferase family 1 member A1; UTR, untranslated region.
