# Supplementary material for: Single nucleotide polymorphisms associated with elevated alanine aminotransferase in patients receiving asunaprevir plus daclatasvir combination therapy for chronic hepatitis C
Source: PLoS One. 2019 Jul 10;14(7):e0219022. doi: 10.1371/journal.pone.0219022 (PMC6619746; doi:10.1371/journal.pone.0219022)
Supplement: S3 Table — (DOCX) [file pone.0219022.s003.docx]

**S3 Table.** Maximum ALT values in each tag SNP genotype of *CYP3A4*, *CYP3A5*, *OATP2B1*, *OATP1B1*, *P-gp*, and *UGT1A1*

| Tag SNP | Genotype | *n* | Maximum ALT value | P value |
| --- | --- | --- | --- | --- |
| ***CYP3A4*** |  |  |  |  |
| rs3735451 | TT/non-TT | 105/80 | 29 (9–561) / 31 (8–740) | 0.897 |
| rs2246709 | AA/non-AA | 79/106 | 30 (9–561) / 29 (8–740) | 0.818 |
| rs4646437 | CC/non-CC | 157/28 | 31 (9–740) / 22 (8–71) | 0.013 |
| ***CYP3A5*** |  |  |  |  |
| rs776746 | GG/non-GG | 113/72 | 31 (9–561) / 26 (8–740) | 0.315 |
| ***OATP1B1*** |  |  |  |  |
| rs4149087 | GG/non-GG | 94/91 | 28 (9–470) / 33 (8–740) | 0.678 |
| rs4149064 | AA/non-AA | 80/105 | 29 (9–470) / 32 (8–740) | 0.668 |
| rs4149048 | AA/non-AA | 76/109 | 30 (9–347) / 30 (8–740) | 0.962 |
| rs7969341 | AA/non-AA | 77/108 | 32 (9–561) / 25 (8–740) | 0.261 |
| rs6487213 | TT/non-TT | 81/104 | 35.5 (8-740) / 25 (9-561) | 0.179 |
| ***OATP2B1*** |  |  |  |  |
| rs4944992 | TT/non-TT | 53/132 | 28 (8–740) / 31 (9–347) | 0.839 |
| rs2712819 | TT/non-TT | 81/104 | 29 (9–179) / 30 (8–740) | 0.832 |
| rs11236365 | AA/non-AA | 59/126 | 30 (9–470) / 29 (8–740) | 0.782 |
| ***P-gp*** |  |  |  |  |
| rs1202168 | TT/non-TT | 71/114 | 28 (9–561) / 31 (8–740) | 0.468 |
| rs1922241 | CC/non-CC | 86/99 | 32 (9-740) / 25 (8-470) | 0.192 |
| rs7779562 | CC/non-CC | 72/113 | 24.5 (9-561) / 33 (8-740) | 0.081 |
| r1858923 | TT/non-TT | 63/122 | 30 (9-740) / 29.5 (8-347) | 0.482 |
| rs868755 | CC/non-CC | 72/113 | 30 (8–740) / 30 (9–347) | 0.628 |
| ***UGT1A1*** |  |  |  |  |
| rs4148323 | GG/non-GG | 132/53 | 28 (8-561) / 33 (9-740) | 0.543 |

Data of maximum ALT values are expressed as medium (range).

SNP, single nucleotide polymorphism; *CYP3A4*, cytochrome P450 family 3 subfamily A member 4; *CYP3A5*, cytochrome P450 family 3 subfamily A member 5; *OATP2B1*, organic anion transporting polypeptide 2B1; *OATP1B1*, organic anion transporting polypeptide 1B1; *P-gp*, P-glycoprotein; *UGT1A1*, uridine diphosphate glucuronosyltransferase family 1 member A1.
