# Supplementary material for: Single nucleotide polymorphisms associated with elevated alanine aminotransferase in patients receiving asunaprevir plus daclatasvir combination therapy for chronic hepatitis C
Source: PLoS One. 2019 Jul 10;14(7):e0219022. doi: 10.1371/journal.pone.0219022 (PMC6619746; doi:10.1371/journal.pone.0219022)
Supplement: S4 Table — (DOCX) [file pone.0219022.s004.docx]

**S4 Table.** Baseline factors associated with ALT elevation (grade ≥1)

| Variable | Category | Univariate analysis |  | Multivariate analysis | |
| --- | --- | --- | --- | --- | --- |
|  |  | p value |  | Odds ratio (95% CI) | p value |
| Age (years) | 1: ≥72  2: <72 | 0.860 |  |  |  |
| Gender | 1: male  2: female | 0.074 |  |  |  |
| Body weight (kg) | 1: ≥55.9  2: <55.9 | 0.710 |  |  |  |
| Body mass index (kg/m^2^) | 1: ≥22.5 | 0.856 |  |  |  |
|  | 2: <22.5 |  |  |  |  |
| Cirrhosis | 1: absence | 0.043 |  | 1.88 (1.01–3.50) | 0.045 |
|  | 2: presence |  |  |  |  |
| rs4646437 genotype | 1: non-CC  2: CC | 0.045 |  | 2.83 (1.05–7.07) | 0.040 |
| Platelet count (×10^4^/μL) | 1: ≥13.0  2: <13.0 | 0.118 |  |  |  |
| AST (IU/L) | 1: <46  2: ≥46 | 0.070 |  |  |  |
| ALT (IU/L) | 1: <40  2: ≥40 | 0.252 |  |  |  |
| γ-GTP (IU/L) | 1: <39  2: ≥39 | 0.586 |  |  |  |
| Albumin (g/dL) | 1: ≥4.0  2: <4.0 | 0.531 |  |  |  |
| Total bilirubin (mg/dL) | 1: ≥0.69  2: <0.69 | 0.207 |  |  |  |
| Creatinine (mg/dL) | 1: ≥0.75  2: <0.75 | 0.234 |  |  |  |
| α-fetoprotein (ng/mL) | 1: <5.9  2: ≥5.9 | 0.088 |  |  |  |
| HCV RNA (log IU/mL) | 1: <6.2  2: ≥6.2 | 0.393 |  |  |  |
| FIB-4 index | 1: <3.92  2: ≥3.92 | 0.168 |  |  |  |

CI, conﬁdence interval; AST, aspartate aminotransferase; ALT, alanine aminotransferase; γ-GTP, γ-glutamyltransferase; HCV, hepatitis C virus; FIB, fibrosis.
