# Supplementary material for: Single nucleotide polymorphisms associated with elevated alanine aminotransferase in patients receiving asunaprevir plus daclatasvir combination therapy for chronic hepatitis C
Source: PLoS One. 2019 Jul 10;14(7):e0219022. doi: 10.1371/journal.pone.0219022 (PMC6619746; doi:10.1371/journal.pone.0219022)
Supplement: S5 Table — (DOCX) [file pone.0219022.s005.docx]

**S5 Table.** Factors associated with sustained virological response

| Variable | Category | Univariate analysis |  | Multivariate analysis | |
| --- | --- | --- | --- | --- | --- |
|  |  | P value |  | Odds ratio (95% CI) | P value |
| Age (years) | 1: ≥72  2: <72 | 0.801 |  |  |  |
| Gender | 1: female  2: male | 0.757 |  |  |  |
| Body weight (kg) | 1: ≥55.9  2: <55.9 | 0.326 |  |  |  |
| Body mass index (kg/m^2^) | 1: ≥22.5  2: <22.5 | 0.795 |  |  |  |
| Cirrhosis | 1: presence  2: absence | 0.030 |  | 3.09 (1.20–7.99) | 0.020 |
| rs4646437 genotype | 1: non-CC  2: CC | 0.527 |  |  |  |
| Platelets count (×10^4^/μL) | 1: <13.0  2: ≥13.0 | 0.688 |  |  |  |
| AST (IU/L) | 1: <46  2: ≥46 | 0.852 |  |  |  |
| ALT (IU/L) | 1: <40  2: ≥40 | 0.445 |  |  |  |
| γ-GTP (IU/L) | 1: <39  2: ≥39 | 0.970 |  |  |  |
| Albumin (g/dL) | 1: ≥4.0  2: <4.0 | 0.534 |  |  |  |
| Total bilirubin (mg/dL) | 1: ≥0.69  2: <0.69 | 0.744 |  |  |  |
| Creatinine (mg/dL) | 1: <0.75  2: ≥0.75 | 0.665 |  |  |  |
| α-fetoprotein (ng/mL) | 1: ≥5.9  2: <5.9 | 0.633 |  |  |  |
| HCV RNA (log IU/mL) | 1: ≥6.2  2: <6.2 | 3.51×10^-4^ |  | 9.25 (3.07–27.86) | 7.68×10^-5^ |
| NS5A L31 substitution | 1: yes  2: no | 0.818 |  |  |  |
| NS5A Y93 substitution | 1: yes  2: no | 1.22×10^-3^ |  | 7.34 (1.92–28.14) | 3.61×10^-3^ |
| Maximum ALT elevation | 1: grade <2  2: grade ≥2 | 0.150 |  |  |  |
| FIB-4 index | 1: <3.92  2: ≥3.92 | 0.801 |  |  |  |

CI, conﬁdence interval; AST, aspartate aminotransferase; ALT, alanine aminotransferase; γ-GTP, γ-glutamyltransferase; HCV, hepatitis C virus; NS5A, non-structural 5A; FIB, fibrosis.
