# Supplementary material for: Single nucleotide polymorphisms associated with elevated alanine aminotransferase in patients receiving asunaprevir plus daclatasvir combination therapy for chronic hepatitis C
Source: PLoS One. 2019 Jul 10;14(7):e0219022. doi: 10.1371/journal.pone.0219022 (PMC6619746; doi:10.1371/journal.pone.0219022)
Supplement: S6 Table — (DOCX) [file pone.0219022.s006.docx]

**S6 Table.** Correlation of serum asunaprevir and daclatasvir concentrations with other quantitative variables on Spearman’s rank correlation coefficient

| Variable | Asunaprevir | |  | Daclatasvir | | |
| --- | --- | --- | --- | --- | --- | --- |
|  | *r*_s_ | P value |  | *r*_s_ | | P value |
| Asunaprevir | 1.000 | – |  | | 0.635 | 4.21×10^-5^ |
| Daclatasvir | 0.635 | 4.21×10^-5^ |  | | 1.000 | – |
| Age | -0.234 | 0.175 |  | | -0.195 | 0.263 |
| Body weight | 0.186 | 0.293 |  | | 0.144 | 0.416 |
| Body mass index | 0.341 | 0.052 |  | | 0.271 | 0.128 |
| Platelets count | 0.008 | 0.966 |  | | 0.120 | 0.492 |
| AST | -0.003 | 0.989 |  | | -0.025 | 0.885 |
| ALT | 0.146 | 0.404 |  | | 0.093 | 0.594 |
| γ-GTP | 0.006 | 0.972 |  | | 0.154 | 0.376 |
| Albumin | 0.024 | 0.891 |  | | -0.089 | 0.611 |
| Total bilirubin | 0.043 | 0.806 |  | | -0.256 | 0.138 |
| Creatinine | -0.095 | 0.588 |  | | 0.031 | 0.860 |
| α-fetoprotein | 0.072 | 0.686 |  | | -0.095 | 0.594 |
| HCV RNA | 0.256 | 0.143 |  | | 0.216 | 0.169 |
| FIB-4 index | -0.220 | 0.204 |  | | -0.228 | 0.188 |

Spearman’s correlation was used to evaluate correlation between serum asunaprevir and daclatasvir concentrations and other quantitative variables. r_s_, Spearman's rho; AST, aspartate aminotransferase; ALT, alanine aminotransferase; γ-GTP, gamma-glutamyltransferase; HCV, hepatitis C virus; FIB, fibrosis.
