# Supplementary material for: Single nucleotide polymorphisms associated with elevated alanine aminotransferase in patients receiving asunaprevir plus daclatasvir combination therapy for chronic hepatitis C
Source: PLoS One. 2019 Jul 10;14(7):e0219022. doi: 10.1371/journal.pone.0219022 (PMC6619746; doi:10.1371/journal.pone.0219022)
Supplement: S7 Table — (DOCX) [file pone.0219022.s007.docx]

**S7 Table.** Comparison of serum asunaprevir and daclatasvir concentrations between two categories in each variable

| Variable | Category | *n* | Asunaprevir | | | Daclatasvir | |
| --- | --- | --- | --- | --- | --- | --- | --- |
|  |  |  | serum concentrations | P value | serum concentrations | | P value |
| Gender | female/male | 19/16 | 370 (34.7-5170) /  397.5 (23.8-1560) | 0.487 | 824 (205-2830) /  633.5 (179-1780) | | 0.476 |
| Cirrhosis | yes/no | 11/24 | 455 (62.1–5170) /  361 (23.8–2020) | 0.522 | 530 (223-1470) /  797.5 (179-2830) | | 0.776 |
| rs4646437 | CC/non-CC | 30/5 | 398.5 (23.8–5170) /  352 (57.5–1200) | 0.888 | 823 (179-2830) /  349 (263-1780) | | 0.571 |
| NS5A L31 substitution | yes/no | 0/26 | - / 435 (23.8–5170) | - | - / 823 (179-2830) | | - |
| NS5A Y93  substitution | yes/no | 1/25 | 78.9 / 443  (23.8-5170) | - | 295 / 824  (179-2830) | | - |
| Max ALT | grade <1/≥1 | 18/17 | 167 (23.8–1660) /  475 (94.3–5170) | 0.023 | 410.5 (205-1780) /  887 (179-2830) | | 0.137 |
| Max ALT | grade <2/≥2 | 25/10 | 352 (23.8–2020) /  536.5 (94.3–5170) | 0.144 | 771 (179-2830) /  812 (223-1590) | | 0.411 |
| Max ALT | grade <3/≥3 | 31/4 | 370 (23.8-2640) /  490 (233-5170) | 0.300 | 737 (179-2830) /  894.5 (530-1470) | | 0.312 |

Data of serum asunaprevir and daclatasvir concentrations are expressed as medium (range).

NS5A, non-structural 5A; ALT, alanine aminotransferase.
